# Honey Bee Location- and Time-Linked Memory Use in Novel Foraging Situations: Floral Color Dependency

**DOI:** 10.3390/insects5010243

**Published:** 2014-02-14

**Authors:** Marisol Amaya-Márquez, Peggy S. M. Hill, Charles I. Abramson, Harrington Wells

**Affiliations:** 1 Institute of Natural Sciences, National University of Columbia, Bogotá, Columbia; E-Mail: mamayam@unal.edu.co; 2 Department of Biological Science, University of Tulsa, Tulsa, OK 74104, USA; E-Mail: peggy-hill@utulsa.edu; 3 Department of Psychology, Oklahoma State University, Stillwater, OK 74078, USA; E-Mail: charles.abramson@okstate.edu

**Keywords:** *Apis mellifera*, context, floral constancy, foraging, honey bee, search image, maximum likelihood, Bayesian

## Abstract

Learning facilitates behavioral plasticity, leading to higher success rates when foraging. However, memory is of decreasing value with changes brought about by moving to novel resource locations or activity at different times of the day. These premises suggest a foraging model with location- and time-linked memory. Thus, each problem is novel, and selection should favor a maximum likelihood approach to achieve energy maximization results. Alternatively, information is potentially always applicable. This premise suggests a different foraging model, one where initial decisions should be based on previous learning regardless of the foraging site or time. Under this second model, no problem is considered novel, and selection should favor a Bayesian or pseudo-Bayesian approach to achieve energy maximization results. We tested these two models by offering honey bees a learning situation at one location in the morning, where nectar rewards differed between flower colors, and examined their behavior at a second location in the afternoon where rewards did not differ between flower colors. Both blue-yellow and blue-white dimorphic flower patches were used. Information learned in the morning was clearly used in the afternoon at a new foraging site. Memory was not location-time restricted in terms of use when visiting either flower color dimorphism.

## 1. Introduction

Many insect nectivores experience changing floral composition in resource patches over their foraging life [1]. Further, this changing composition of floral resources available to pollinators is not restricted to seasonal events. The floral landscape for a nectivore can change many times during a single day due to the endogenous patterns of nectar secretion and pollen presentation of angiosperms [2,3,4], as well as weather and the activity of other pollinators [5,6]. Thus, even relatively short-lived foragers like honey bees face changes in nectar and pollen sources that have different reward potentials and associated costs. This situation creates a fundamental problem for foragers.

Learning allows foragers to adapt to their changing environment through behavioral plasticity [7,8,9]. For example, bumble bees can learn to estimate interval duration just as vertebrates do [10], which enables them to exploit resources in a complex foraging environment in their well-known trap-line behavior [11,12,13,14,15]. Further, a connection may exist between learning and fitness of foragers (e.g., [16,17,18], but see [19]). In diverse species individuals that are able to associate cues with reward quality achieved higher growth rates [20] and/or greater food caches [21] that would translate into survival and reproductive fitness. For example, in a comparative study of bumble bees, the fastest learning colonies collected 40% more nectar per unit time than the slowest learners [18]. Further, useful memory information can be applied under appropriate circumstances to solve new problems, and problem solution through analogy is both a noted mark of intelligence and a benefit of memory [22]. 

On the other hand, the decreasing value of recently acquired information when environmental change occurs is a cost of memory [23,24]. An additional cost is incurred when memory actually interferes with subsequent learning [25,26], so that a search image leads to floral fidelity that is sub-optimal [27,28,29,30] and residual memory modifies forager distribution among patches from that of the ideal free distribution [31]. In addition, the ability to learn has evolutionary trade-offs with other fitness-related traits [26,32]. Faster-learning *Drosophila* not only show lower larval competitive ability when they are not required to use learning [33] but also lowered fecundity of adults under conditions when learning was required [19]. Africanized honey bees, which continue to expand their range and displace the European honey bee, are not competitive with the European honey bee in learning and memory tests of associating odor with reward [34,35,36]. Thus, the more complete and perfect the process of memorization, the higher can be the cost of cognition. 

Clearly, through learning honey bees visiting a flower patch are able to maximize net energy gain through choices that increase calories consumed [37,38,39,40,41,42,43], reduce flight time between flowers [44,45] and minimize flower handling time [46,47]. How they use this acquired information when addressing a novel foraging situation is uncertain. Two fundamental cognitive approaches exist [48].

The first approach is that foraging memory is space-time linked. If so, different locations are treated as new problems, and the initial response is to sample alternative flowers following maximum likelihood principles. The maximum likelihood approach assumes that the forager, when approaching a new problem, uses no prior knowledge of alternative rewards, cues associated with those rewards or energetic costs involved. The sample estimated reward and cost values are used as if they are the true values, since it is the maximum likelihood estimate of those true parameters [49]. 

Alternatively, an approach to a novel foraging situation can consider memory information as applicable to all situations regardless of time or location. Different locations are thus not treated as new problems, and the initial response is to base foraging on previously learned relationships until information to the contrary is obtained, which is a Bayesian approach [48,49]. Here, the forager approximates the initial distribution of possible results associated with alternative flower choices based on prior learned information from completely different foraging situations, and continually updates estimates as it forages [48,49]. This seems cognitively possible for honey bees based on the work of Naug and Arathi [50] and Sanderson *et al.* [51] when presenting bees with novel situations at the same location. Key to the Bayesian approach for foragers is that there is some prior expectation about the frequency of each potential net reward associated with specific floral cues. 

Here we report on whether foragers restrict use of reward information linked to a specific time and location in the environment (space-time linked memory), and thus use a maximum likelihood “new problem approach”, or whether they apply information learned earlier in different contexts (different location and time) to new situations in a Bayesian type of strategy.

## 2. Experimental Section

Italian honey bees exhibit very different behaviors depending on the flower colors offered them as cues in the experimental design of two-choice foraging tests. When presented with a blue-yellow dimorphic flower patch where both choices are rewarded, some foragers will exhibit fidelity to blue flowers and others to yellow flowers, even when one color provides a greater reward [39,52]. In contrast, if a patch contains equally rewarding blue and white flowers, individuals will forage randomly with respect to color and so visit both colors extensively. The same honey bees will immediately switch to forage on one color of flower when rewards are unequal in blue-white dimorphic flower patches. This blue-yellow/blue-white anomaly has been characterized as a context-dependent behavior [45,47,52,53]. Thus, we tested bees using both blue-yellow and blue-white dimorphic flower patches to investigate and attempt to control these intrinsic constraints to foraging choices of unrestrained bees.

The behavior of foraging Italian honey bees on patches of blue and yellow flowers does not appear to be a result of adaptation to the environment in a specific geographic region [45,47,51,52], which we have recognized as a confounding factor in studies of congeneric species (e.g., [54]). Studies using a caged hive populated by only newly eclosed, and therefore environmentally naive, bees show the same behavioral response where some individuals only visit blue flowers and others yellow flowers [55]. Further, when the artificial flowers are grouped in pairs and the pairs spaced farther apart (meters), honey bee behavior remains the same. A bee constant to yellow, for example, will fly to the next pair to again visit a yellow rather than visit the blue flower just centimeters away, which is quite different than the behavior of bees visiting blue and white flowers [45]. The general competition model developed by Levin and Anderson [56] assumes that pollinators always visit the nearest plant while foraging, and this is not the case for Italian honey bees.

Foragers appear to differentiate both blue from white and blue from yellow quite well based on fidelity level to a flower color when rewards differ between flower colors [39,47,57]. In addition, Lehrer and Bischof [58] determined that honey bees discriminate easily between yellow and blue/violet pigments. This conclusion is further supported by the fact that many times it takes just visitation to three flowers on a trip to a flower patch to switch fidelity from one color to another on patches containing blue and white flowers [52]. The blue-yellow response observed on artificial flower patches has also been observed in agricultural settings [59,60,61,62,63], and so is not simply an artifact of the artificial flower system. 

The honey bee’s neurological pathway when dealing with blue-white *versus* blue-yellow flower choices appears to be fundamentally different. Under the influence of ethanol (<5%) honey bee foragers lose their fidelity for the flower color offering the higher-caloric reward when choosing between blue and white flowers; when intoxicated each bee randomly visits flowers with respect to color. In contrast, forager fidelity for flower color remains unchanged when choosing between blue and yellow flowers; some bees visit only blue and the rest only yellow flowers when intoxicated [64]. 

The evolutionary underpinnings may have to do with division of labor in a large-colony eusocial species. Although energy maximization on an individual forager basis is not always met, fitness is based on the colony as a whole. Here, including nectar collection from less profitable resources may provide an advantage in what might be likened to a “scorched earth” policy where little is left for competitors. Even so, foragers must recognize that specific flowers in a patch have been pollinated and thus are no longer productive nectar sources. Pollination is often accompanied by a change in flower color towards pallid [3,65]. 

In contrast to the division-of-labor/competition theory, Abramson suggests that honey bees accomplish many of the tasks once thought to be limited to advanced vertebrates in very different cognitive manners [66,67]. Supporting that argument is the fact that honey bees do not respond to the removal of a stimulus as a conditioning cue [68]. Under this model, the reward-independent flower color fidelity observed when bees are visiting flower patches of blue and yellow flowers may be a non-selective side effect of the means honey bees employ to solve other problems with only 1% of the neurons of animals like rodents and only 0.1% of those in of house cats [69]. However, bumble bees don’t exhibit the blue-yellow type of flower constancy observed in honey bees [70,71]. In fact, in a direct comparison of foraging with respect to color, Gegear and Laverty found, “*All honey bees showed a high degree of flower constancy to one colour and rarely visited the alternate colour, whereas most bumble bees indiscriminately visited both colours*” [70]. This at least begs the question to be explored further: whether colony size has been a factor in the evolution of this behavior, as suggested by the division-of-labor/competition theory? 

The details of an experimental flower patch and the level of interaction between the experimenter and the forager can confound levels of flower color constancy. We have concluded that even differences in materials or methods that appear to be minute can be sufficient to elicit differences in behavior in honey bees. This includes whether experimental design allows foragers to walk between flowers, simultaneous or sequential rewards are associated with the CS (conditioned stimulus), and forced sampling even occurs [52,72]. These factors, for example, led to very different views of honey bee floral color-constancy by Banschbach [73] and Hill *et al.* [52], and are exacerbated by Banschbach’s failure to switch the more rewarding choice to the alternative color in a reverse experiment. Italian honey bees that are free-flying, cannot walk between flowers, and given simultaneously presented rewarded choices of yellow and blue flowers (even if rewards are not identical in the two colors) will continue to visit the flower color they first visited upon arrival at the flower patch as long as some reward is provided. They may never sample the alternative floral color, but even if the alternative color choice contains a reward that is richer in caloric value (greater sugar concentration, greater volume, or even higher frequency of reward) foragers remain faithful to their initial flower color choice [38,39,45,47]. 

Further, even Darwin observed that both honey bees and bumble bees are not confused by the variation of color hues found in a natural patch of flowers of a single species [74]. Chittka showed us 20 years ago that the spectral properties of flowers excite a honey-bee’s three color receptors in a way that leads to groupings of similar colors in the bee brain’s color-space [75]. Yet, some conclusions about color constancy ignore differences in colors that are distinct to human eyes, but that bees perceive as similar [52]. For example, in testing reward volume influences on honey bee foraging choices, Greggers and Menzel [41] found that constancy to floral color declined when the reward volume was reduced, but they tested using human-white and violet. 

**Figure 1:**
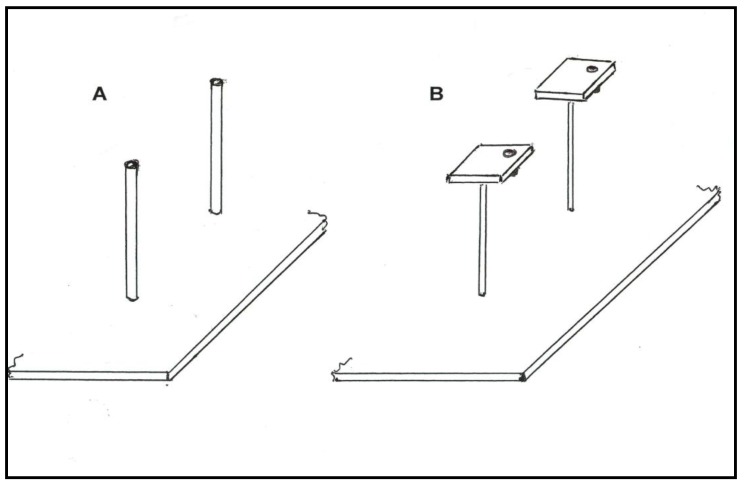
Flower morphologies used in experiments. Tube flowers (**A**) have a false bottom 15 mm from the top, which makes the well-depth the same as that of flat flowers. Flat flowers (**B**) have the same well opening to enter and same well depth for the reward as the tube flowers. Thus, no difference in effort needed to obtain rewards from the alternative flower morphologies existed (Panel A and B drawn by H.W.).

All experiments used free-flying honey bees, *Apis mellifera ligustica*, foraging outdoors on artificial flower patches of 36 flowers spaced 75 mm a part in rows and columns of a 6 × 6 Cartesian coordinate system on a brown pegboard. Individual bees were given a coded pattern of dots of enamel paint on the thorax for quick recognition. Two different flower designs (flat or tube) were used in the experiments; however, only one shape was used in a flower patch at a given time. Tube flowers consisted of an 8 mm diameter (ID) Plexiglas tube, 90 mm long with a false bottom 15 mm from the flower’s mouth. Tube flowers did not have pedicels and were oriented perpendicular to the pegboard surface. Flat flowers consisted of a 28 mm × 28 mm Plexiglas square, 6 mm thick, with a 8 mm diameter (internal diameter: ID), 15 mm deep Plexiglas tube inserted into one corner that held the reward. Each flower was mounted on a 90 mm pedicel of 5 mm doweling and oriented in a position horizontal to the pegboard surface (Figure 1). 

The color of a flower was blue, yellow or white. Flower patches consisted of either blue and white or blue and yellow flowers in equal number, randomly arranged with respect to color and position within the array. Flowers of different colors were created by painting the lower surface of all flowers and the outer surface of the Plexiglas tube with blue, yellow or white enamel paint (Testors^TM^ paint Nos. 1208 blue, 1214 yellow, and 1245 white). The reflectance spectra for the paints, and a color hexagon depicting how these colors are perceived by the honey bee, can be found in Hill *et al.* [52]. Four experiments were performed.

**Figure 2:**
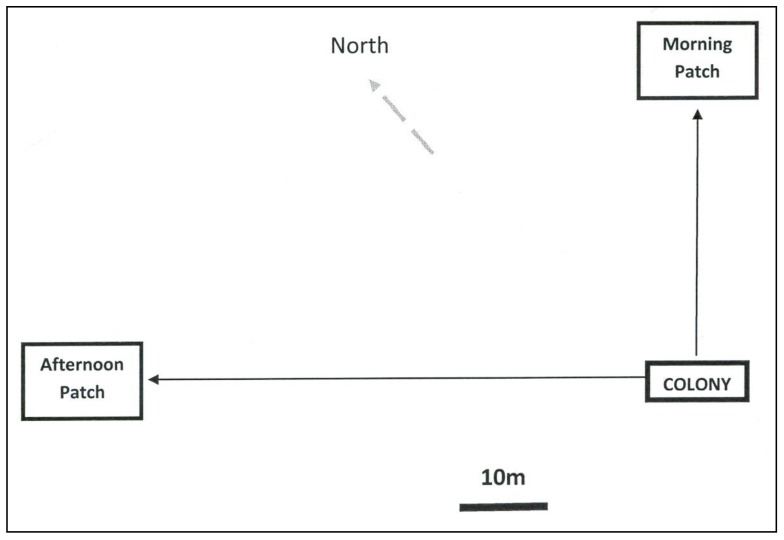
Scheme of geographic layout of the experiment giving the location of the honey bee colony, and experimental sites where flower patches were located (drawn by H.W.).

Free-flying honey bees were trained to two feeding locations (1 and 2) for Experiments I–IV. Site 1 was 30 m from the hive and only offered rewards in the morning (900 to 1,100 h Central Daylight Time (CDT)). Site 2 was 60 m from the hive and only offered rewards in the afternoon (1,600 to 1,800 h CDT). The two sites were located 90 degrees apart with respect to the hive: Site 1 was NE and Site 2 NW of the hive (Figure 2). Individuals were trained to visit clear, colorless petri dishes with 1.5 M sucrose solution scented with 5 μL/L clove oil at Site 1 and 5 μL/L peppermint oil at Site 2. Training to the sites was done over a period of two days. There was no training to color. The first experimental exposure to test colors occurred when an artificial flower patch, on which bees were allowed to forage freely, was introduced to replace the petri dish at the feeding site during the specific reward periods. A different set of bees was used for each experiment. All bees in a given experiment experienced each treatment of the experiment for approximately one hour. Flowers were washed in unscented detergent and then triple rinsed and allowed to dry after each use.

Most bees trained to the morning experimental site did not visit the afternoon location (about 10% visited both). This is probably not surprising since foragers visiting the morning site could very well be committed already to one or more afternoon sites (crop attached to some other afternoon site) as shown by Wagner *et al.* [76].

Bees were free to leave and return to the colony at will, and navigated to and from the experimental sites on their own accord. Consequently, foragers in the experiments had varied prior experiences with cues associated rewards, including colors and scents. 

Individual bees were uniquely marked by a dot of Testors^®^ paint on the thorax. Those not used in a trial were kept occupied with a “super feeder” 10 m from the flower patch. Any intruder bees were removed from the system. Individuals were allowed to freely choose flowers to visit. The flower sequence that each bee visited during a visit to the flower patch was recorded by an observer. A visit occurred (was recorded by an observer) when a bee completely entered the nectar well to taste the reward offered by a flower, whether or not the reward was consumed. A flower was refilled by the observer when a bee made the next flower choice. Bees foraging on flowers paid no noticeable attention to the observers.

Four experiments were performed (Table 1). Each experiment used from 14 to 29 bees that experienced all three treatments (*experimental bees* visiting both morning and afternoon sites) and 11 to 23 bees that received only Treatment 3 (*control bees* that were not visiting the morning site). Specific numbers of bees used are given with the statistical results for each experiment. Three to four bees were followed in a trial of an experiment: typically 2 experimental and 1 or 2 control bees. Bee interaction was not an issue because time spent unloading at the hive was about 7 to 8 times longer than foraging on the patch, which meant that usually only one bee was on the flower patch at a time. A new set of bees was used for each trial of every experiment. One trial was performed per day. Experimental bees each made 14 to 15 trips from the hive over a 3 treatment experiment, and control bees 4 to 5 trips since they only participated in Treatment 3.

### 2.1. Blue and Yellow Flowers: Reward Odor Differing between Sites

The experiment had three treatments. Treatments 1 and 2 were performed at Site 1 in the morning and Treatment 3 at Site 2 in the afternoon. Both morning and afternoon flower patches contained 18 blue and 18 yellow “flat” flowers randomly arranged with respect to color. Both flower colors offered foragers 4 μL of 1 M sucrose with 5 μL/L clove oil in Treatment 1. Foragers were offered the choice of 4 μL of 2 M *versus* 4 μL of 1 M sucrose (both scented with 5 μL/L clove oil) in the alternative flower colors in Treatment 2, with approximately half of the bees offered blue flowers that held the greater reward and the other half offered yellow flowers with the greater reward. Nectar rewards in Treatment 3 were the same as in Treatment 1 except that the nectar odor was 5 μL/L peppermint oil. Thus time, location and odor varied in Treatment 3, while color and shape were the same in all three treatments. Reward quality (molarity) did not differ among the two choices in Treatment 3 as it had in Treatment 2. Flower choice by a set of bees that had not visited the morning patch was also recorded as an experimental control in Treatment 3 to test whether exposure to Treatments 1 and 2 altered behavior of the bees in the afternoon.

**Table 1:** Summary of treatments in each experiment. In Treatment 2, one of the two flower colors offered 2 M sucrose reward and the other 1 M sucrose reward. Half of the trials of each experiment were with blue flowers offering the 2 M reward, and the other half of the trials were with the alternative flower color offering the 2 M reward.

Exp. #	Flower Colors	Morning						Afternoon		
		Site 1						Site 2		
		Treatment 1			Treatment 2			Treatment 3		
		Rewards	Odor	Flower Shape	Rewards	Odor	Flower Shape	Rewards	Odor	Flower Shape
**1**	**Blue & Yellow**	1 M both colors	clove	flat	1 M:2 M	clove	flat	1 M both colors	mint	flat
**2**	**Blue & Yellow**	1 M both colors	clove	flat	1 M:2 M	clove	flat	1 M both colors	mint	tube
**3**	**Blue & White**	1 M both colors	clove	flat	1 M:2 M	clove	flat	1 M both colors	mint	flat
**4**	**Blue & White**	1 M both colors	clove	flat	1 M:2 M	clove	flat	1 M both colors	mint	tube

### 2.2. Blue and Yellow Flowers: Flower Shape and Reward Odor Differing between Sites

The experimental design for Experiment II was the same as described for Experiment I except for flower morphology (shape) at Site 2 (afternoon). Morning flower patches contained 18 blue and 18 yellow “flat” flowers randomly arranged with respect to color, while afternoon patches contained 18 blue and 18 yellow “tube” flowers randomly arranged as to color. Thus, flowers shared only color similarity in morning and afternoon sites, while the flower shape and reward odor, as well as location and time, differed from the morning site. Nectar rewards in each treatment were the same as those offered in Experiment I. As in Experiment I, reward quality that had varied between the two color choices in Treatment 2 were the same in Treatment 3. Flower choice by a set of bees that had not visited the morning patch was also recorded as an experimental control to test whether morning treatments had an effect on behavior in the afternoon.

### 2.3. Blue and White Flowers: Reward Odor Differing between Sites

Experiment III repeated Experiment I using blue and white for the flower colors instead of blue and yellow. Morphologically, flowers were the same in morning and afternoon flower patches. However, flowers differed in the odor associated with nectar in the afternoon patch. Thus flower color and shape were the same in all three treatments, while the location, time and reward odor varied in Treatment 3. Reward quality was the same in both colors in Treatments 1 and 3. Flower choice by a set of bees that had not visited the morning patch was also recorded as an experimental control to test whether morning treatments had an effect on behavior in the afternoon.

### 2.4. Blue and White Flowers: Flower Shape and Reward Odor Differing between Sites

Experiment IV repeated Experiment II using blue and white rather than blue and yellow flowers. Flowers in the afternoon patch differed from those in the morning in shape, as well as odor associated with the reward, time and location. Only the color in the two-choice test was the same in all three treatments, and the reward quality was the same in both choices in Treatments 1 and 3. Flower choice by a set of bees that had not visited the morning patch was also recorded as an experimental control to test whether morning treatments had an effect on behavior in the afternoon.

### 2.5. Data Analysis

Forager-type was assigned by the preferred flower color of the first five flowers a bee visited on her first visit to the flower patch (*i.e*., choosing blue or the alternative color, since flower patches were dimorphic). Repeated measures MANOVAs [77] were used to analyze data from Experiments I–IV. Each experiment was analyzed separately based on the arcsine square-root transformation of the relative frequency (proportion) of visits to blue flowers (following [78]). We tested for treatment, forager-type (*i.e*., blue or not-blue bee), association (Treatment 2 with blue flowers offering the higher caloric reward, or with yellow or white flowers offering the greater reward) and interaction effects.

Next, data from control bees (bees that did not visit the morning flower patch and therefore did not have exposure to treatments 1 and 2) was combined with the corresponding data from Treatment 3 of the experimental bees (bees that were exposed to Treatments 1 and 2). ANOVAs [77] were used to analyze these data from Experiments I-IV. Each experiment was analyzed separately based on the arcsine square-root transformation of the relative frequency (proportion) of visits to blue flowers. We tested for forager-type, association (Treatment 2 with blue flowers with a greater caloric reward, or Treatment 2 with yellow or white flowers offering the better reward, or no Treatment 2) and interaction effects.

## 3. Results and Discussion

In all four experiments flower color choice in the afternoon foraging location was highly predictable from the behavior observed at the morning foraging site. Flower color was remembered from the earlier foraging experience and used in the afternoon at the new location. However, use of flower color in blue-yellow *versus* blue-white flower patches provided foragers with a very different context and this shaped how the information was used.

### 3.1. Blue-Yellow Dimorphic Flower Patches

**Figure 3:**
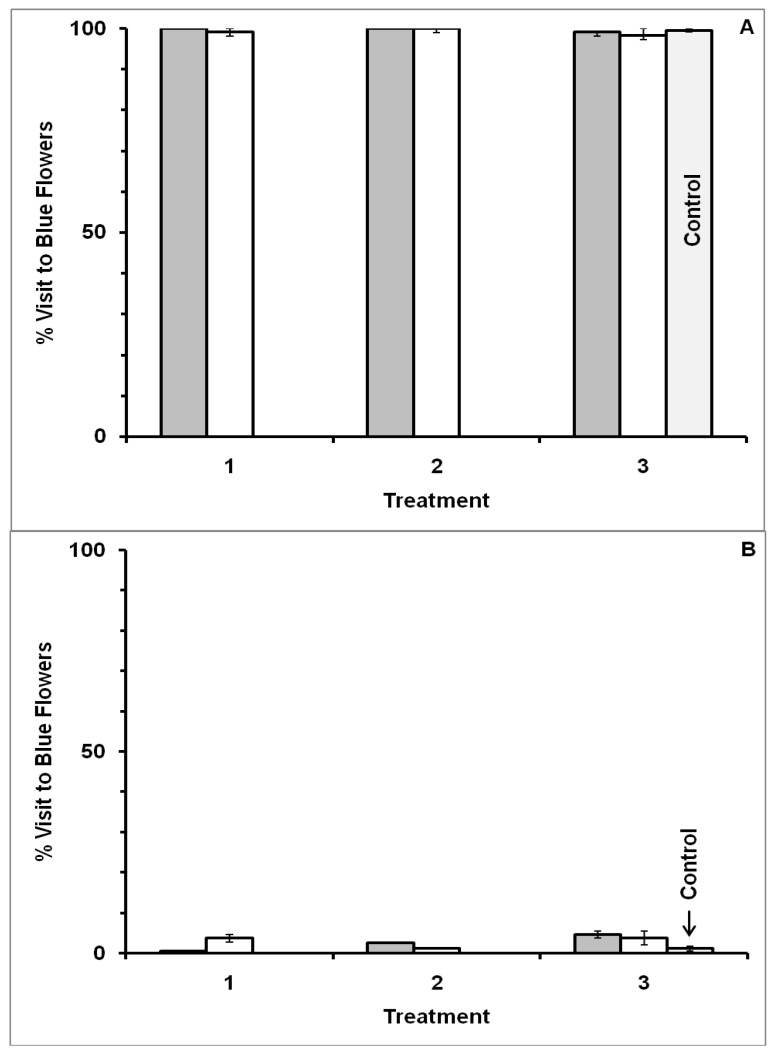
Visitation by bees to blue-yellow dimorphic flower patches in Experiment I. Reward odor, site location and time differed between morning and afternoon flower patches. Percentage of blue flowers visited is presented for blue forager-type bees in Panel (**A**) (N = 7 bees), and for yellow forager-type bees in Panel (**B**) (N = 7 bees). Error bars indicate ±1 SE. Bar color is by flower color offering the higher molar sucrose reward in Treatment 2: gray bars = bees given the higher molar reward in blue flowers, and white bars = bees given the higher molar reward in yellow flowers. Treatment 1: morning patch, flat flowers with clove scent, with both blue and yellow flowers offering the same reward. Treatment 2: morning patch, flat flowers with clove scent, with reward difference between blue and yellow flowers. Treatment 3: afternoon patch, flat flower with peppermint scent, with both blue and yellow flowers offering the same reward. Control represents bees that had never visited the morning patch, and thus never experienced Treatments 1 and 2 (N = 11 bees).

In Experiment I where odor differed as well as location of morning and afternoon flower patches (Figure 3), foragers visiting flat blue-yellow dimorphic flower patches exhibited a significant forager-type effect (MANOVA: F_1,10_ = 1244.7800, *p* < 0.0001), but not an association (F_1,10_ = 0.1030, *p* = 0.7549), treatment (F_2,9_ = 0.0379, *p* = 0.9630) or interaction effect (type × association: F_1,10_ = 0.1302, *p* = 0.7257; type × treatment: F_2,9_ = 2.3020, *p* = 0.1558; association × treatment: F_2,9_ = 1.3721, *p* = 0.3019; type × treatment × association: F_2,9_ = 3.2967, *p* = 0.0843). Bees that initially visited blue flowers in the morning continued to visit blue flowers after a time lag of at least 5 h, and bees that initially visited yellow flowers continued to visit yellow, even when the reward odor, location and time were different in the afternoon in Treatment 3. The reward quality in Treatment 3 did not vary between floral colors and thus no longer supplied the difference in molarity of rewards associated with colors that was present in Treatment 2. Results were based on 14 bees (7 blue forager-type and 7 yellow forager-type) that visited 1,987 flowers in total over the three treatments (flower visited per bee 141.9 ± 1.8: mean ± SE).

**Figure 4:**
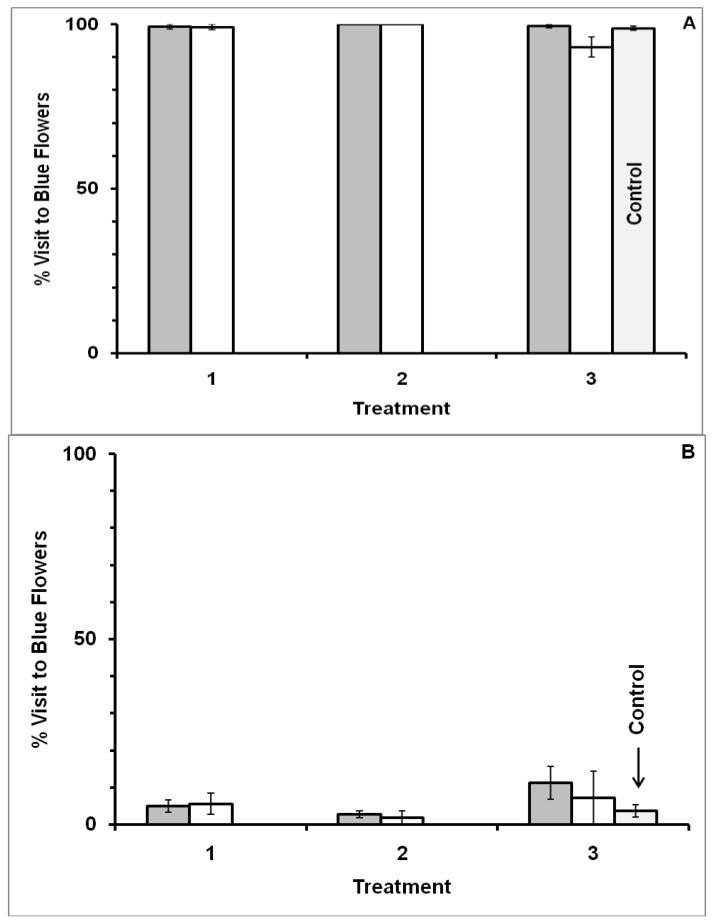
Visitation by bees to blue-yellow dimorphic flower patches in Experiment II. Flower shape as well as reward odor, site location and time differed between morning and afternoon flower patches. Percentage of blue flowers visited is presented for blue forager-type bees in Panel (**A**) (N = 8 bees), and for yellow forager-type bees in Panel (**B**) (N = 7 bees). Error bars indicate ±1 SE. Bar color is by flower color offering the higher molar sucrose reward in Treatment 2: gray bars = bees given the higher molar reward in blue flowers, and white bars = bees given the higher molar reward in yellow flowers. Treatment 1: morning patch, flat flowers with clove scent, with both blue and yellow flowers offering the same reward. Treatment 2: morning patch, flat flowers with clove scent, with reward difference between blue and yellow flowers. Treatment 3: afternoon patch, tube flowers with peppermint scent, with both blue and yellow flowers offering the same reward. Control represents bees that had never visited the morning patch, and thus never experienced Treatments 1 and 2 (N = 12 bees).

The same behavior was observed when flower shape also differed between morning and afternoon blue-yellow dimorphic flower patches (Experiment II). A significant forager-type effect (F_1,11_ = 1115.6248, *p* < 0.0001) was observed (Figure 4), but no association (F_1,11_ = 2.6533, *p* = 0.1316), treatment (F_2,10_ = 0.2117, *p* = 0.8127) or interaction effects (type × association: F_1,11_ = 0.0024, *p* = 0.9618; type × treatment: F_2,10_ = 2.5768, *p* = 0.1251; association × treatment: F_2,10_ = 0.9933, *p* = 0.4041; type × treatment × association: F_2,10_ = 0.1714, *p* = 0.8449). Honey bees that had first visited flat blue flowers continued to visit blue tube flowers after a minimum 5 h delay when reward odor, flower shape, test location and time were different. The change to equal molarity rewards in Treatment 3 did not affect individual fidelity to floral color. Results were based on 15 bees (8 blue forager-type and 7 yellow forager-type) that visited 2,092 flowers in total over the three treatments (flowers visited per bee 139.5 ± 1.6: mean ± SE). 

In both Experiments I and II some bees visited only blue flowers while other bees visited only yellow flowers. Foragers did not respond to the reward differences associated with flower color in Treatment 2 in either experiment. Further, no bee changed flower color fidelity between morning and afternoon flower patches (Figure 3 and Figure 4). Like the bees that visited the morning flower patch, control bees that were new visitors in Treatment 3 to the afternoon patches showed high fidelity to a flower color (type effect) in both Experiments I and II (Figure 3 and Figure 4).

When control bees and the corresponding experimental bees from Treatment 3 were combined for comparison, bees showed a type effect in both Experiment I (ANOVA: F_1,19_ = 894.5421, *p* < 0.0001) and Experiment II (ANOVA: F_1,21_ = 393.3600, *p* < 0.0001). There was no association (association with a greater caloric reward in Treatment 2 to yellow or to blue, or to neither for the control) effect (Experiment I ANOVA: F_2,19_ = 0.4859, *p* = 0.6226; Experiment II ANOVA: F_2,21_ = 1.9460, *p* = 0.1678), or type × association interaction effect (Experiment I ANOVA: F_2,19_ = 0.8528, *p* = 0.4419; Experiment II ANOVA: F_2,21_ = 0.6919, *p* = 0.5117). Results were based on 11 control bees that visited 572 flowers in total (flowers visited per bee 52.0 ± 1.1: mean ± SE) and 14 experimental bees that visited 763 flowers in total (flowers visited per bee 54.5 ± 1.4: mean ± SE) in Treatment 3 of Experiment I, and on 12 control bees that visited 656 flowers in total (flowers visited per bee 54.7 ± 1.7: mean ± SE) and 15 experimental bees that visited 815 flowers in total (flowers visited per bee 54.3 ± 1.8: mean ± SE) in Treatment 3 of Experiment II.

### 3.2. Blue-White Dimorphic Flower Patches

**Figure 5:**
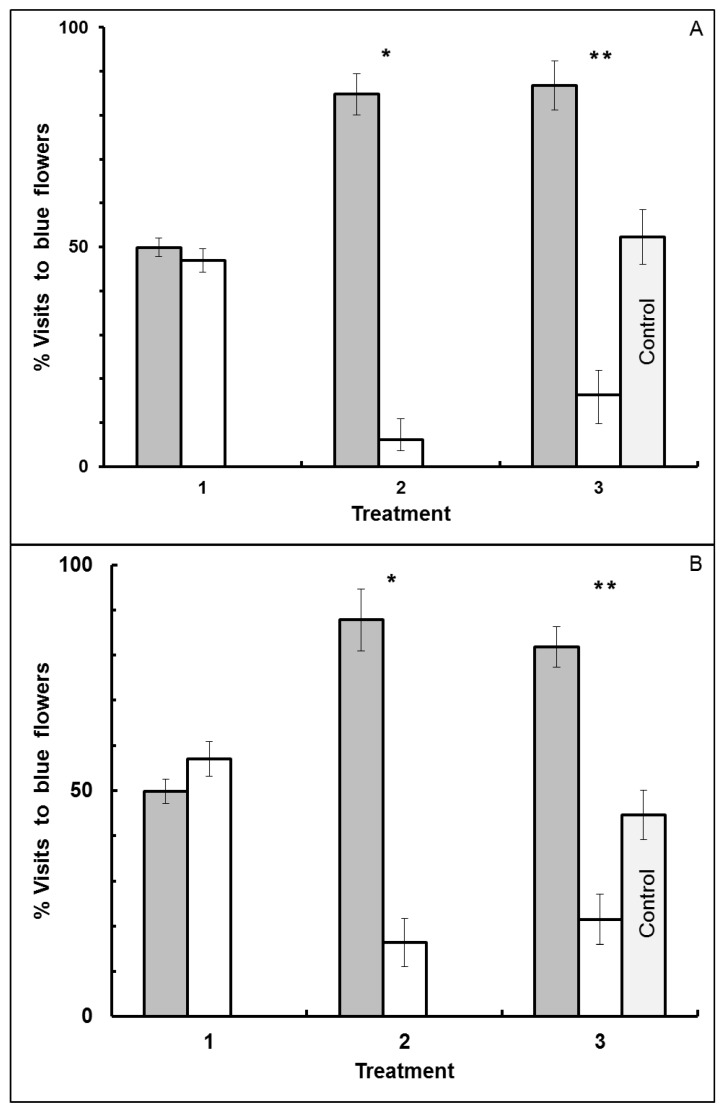
Visitation by bees to blue-white dimorphic flower patches in Experiment III. Reward odor, site location and time differed between morning and afternoon flower patches. Percentage of blue flowers visited is presented for blue forager-type bees in Panel (**A**) (N = 14 bees), and for white forager-type bees in Panel (**B**) (N = 15 bees). Error bars indicate ±1 SE. Bar color is by flower color offering the higher molar sucrose reward in Treatment 2: gray bars = bees given the higher molar reward in blue flowers, and white bars = bees given the higher molar reward in white flowers. Treatment 1: morning patch, flat flowers with clove scent, with both blue and white flowers offering the same reward. Treatment 2: morning patch, flat flowers with clove scent, with reward difference between blue and white flowers. Treatment 3: afternoon patch, flat flowers with peppermint scent, with both blue and white flowers offering the same reward. Control represents bees that had never visited the morning patch, and thus never experienced Treatments 1 and 2 (N = 23 bees). * significant difference (*p* < 0.05).

However, behavior was context-dependent with respect to flower color. Foraging behavior differed from that observed when bees visited blue-yellow dimorphic patches when blue-white dimorphic flower patches were used. Significant association (MANOVA: F_1,25_ = 177.0717, *p* < 0.0001), and treatment × association (F_2,24_ = 85.4544, *p* < 0.0001) occurred, but no forager-type (F_1,25_ = 1.9673, *p* = 0.1730), treatment (F_2,24_ = 0.3497, *p* = 0.7084) or other interaction effects (type × association: F_1,25_ = 2.3425, *p* = 0.1384; type × treatment: F_2,24_ = 0.7225, *p* = 0.4958; type × treatment × association: F_2,24_ = 0.0426, *p* = 0.9583), were observed in Experiment III. Each bee in Treatment 1 extensively visited both blue and white flowers (Figure 5). However, when bees were given the better reward in white flowers in Treatment 2, they quickly learned to favor white flowers, and continued to show fidelity to white flowers in Treatment 3. Similarly, bees given the better reward in blue flowers in Treatment 2 quickly learned to favor blue flowers, and continued to show fidelity to blue flowers in Treatment 3. As in Experiment I, only flower color and shape were the same in all three treatments of Experiment III, while the location, time and reward odor varied in Treatment 3. Reward molarity was the same in both flower colors in Treatments 1 and 3. Results were based on 29 bees (15 blue forager-type and 14 white forager-type) that visited 3,776 flowers in total over the three treatments (flower visited per bee 130.2 ± 1.7: mean ± SE).

**Figure 6:**
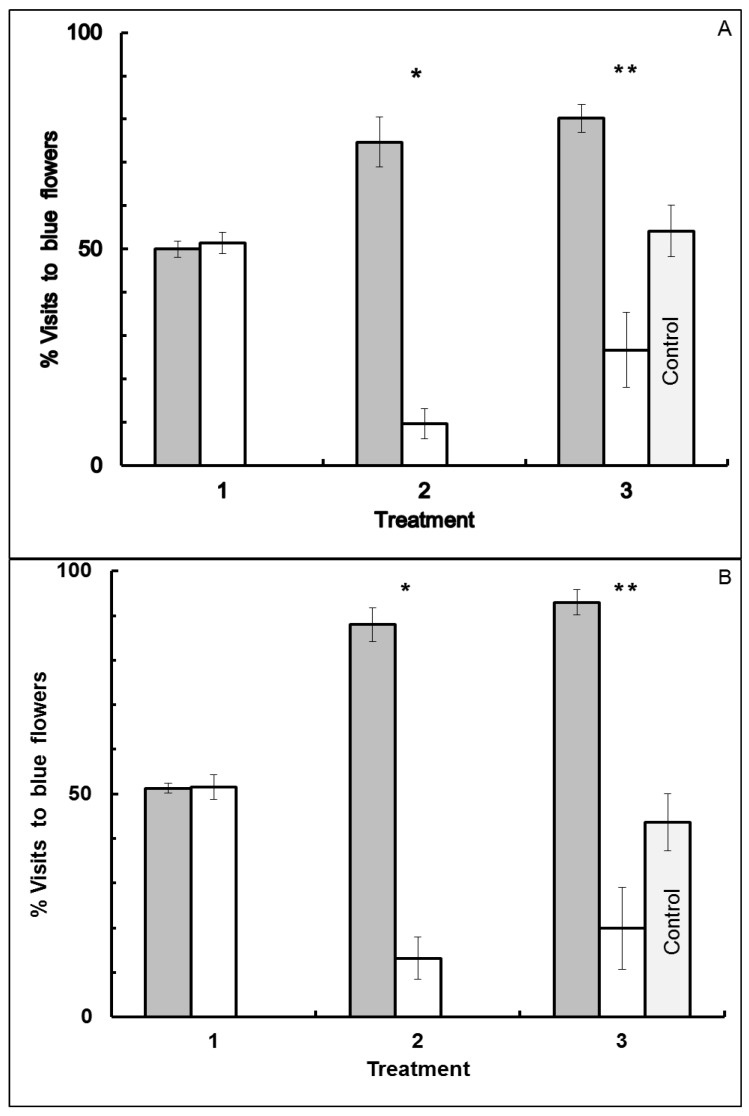
Visitation by bees to blue-white dimorphic flower patches in Experiment IV. Flower shape as well as reward odor, site location and time differed between morning and afternoon flower patches. Percentage of blue flowers visited is presented for blue forager-type bees in Panel (**A**) (N = 12 bees), and for white forager-type bees in Panel (**B**) (N = 13 bees). Error bars indicate ±1 SE. Bar color is by flower color offering the higher molar sucrose reward in Treatment 2: gray bars = bees given the higher molar reward in blue flowers, and white bars = bees given the higher molar reward in white flowers. Treatment 1: morning patch, flat flowers with clove scent, with both blue and white flowers offering the same reward. Treatment 2: morning patch, flat flowers with clove scent, with reward difference between blue and white flowers. Treatment 3: afternoon patch, tube flowers with peppermint scent, with both blue and white flowers offering the same reward. Control represents bees that had never visited the morning patch, and thus never experienced Treatments 1 and 2 (N = 20 bees). * significant difference (*p* < 0.05).

The same behavior was observed in Experiment IV where flower shape also differed between morning and afternoon blue-white flower patches (Figure 6). Significant association (F_1,21_ = 139.4500, *p* < 0.0001) and treatment × association effects (F_2,20_ = 62.2221, *p* < 0.0001), but not a forager-type (F_1,21_ = 1.5797, *p* = 0.2260), treatment (F_2,20_ = 2.7170, *p* = 0.0904) or other interaction effect (type × association: F_1,21_ = 3.4809, *p* = 0.0761; type × treatment: F_2,20_ = 0.9035, *p* = 0.4211; type × treatment × association: F_2,20_ = 1.0496, *p* = 0.3686), were observed in Experiment IV. As in Experiment II, flower shape and odor differed, as well as the site location and time, between morning and afternoon blue-white flower patches. Reward molarity was the same in both flower colors in Treatments 1 and 3 but varied between the flower colors in Treatment 2. Results were based on 25 bees (13 blue forager-type and 12 white forager-type) that visited 3,168 flowers in total over the three treatments (flower visited per bee 126.7 ± 1.7: mean ± SE). 

In both Experiments III and IV flower choice was random with respect to color in Treatment 1 where flower colors offered identical rewards. Individuals visited both colors in this two-choice test. In Treatment 2, bees favored the flower color that offered the higher molarity sucrose reward in both experiments (Figure 5 and Figure 6).

When control bees and the corresponding experimental bees from Treatment 3 were combined for comparison, bees showed an association effect (association in Treatment 2 to blue or white with a higher caloric reward, or to no Treatment 2 for the control) in both Experiment III (ANOVA: F_2,46_ = 40.4350, *p* < 0.0001) and Experiment IV (ANOVA: F_2,39_ = 31.7946, *p* < 0.0001). However, there was no forager-type effect (Experiment I ANOVA: F_1,46_ = 0.2166, *p* = 0.6439; Experiment II ANOVA: F_1,39_ = 0.0386, *p* = 0.8453), or type × association interaction effect (Experiment I ANOVA: F_2,46_ = 0.8161, *p* = 0.4485; Experiment II ANOVA: F_2,39_ = 2.3630, *p* = 0.1075). Results were based on 23 control bees that visited 886 flowers in total (flowers visited per bee 38.4 ± 1.1: mean ± SE) and 29 experimental bees that visited 964 flowers in total in Treatment 3 (flowers visited per bee 33.2 ± 1.6: mean ± SE) of Experiment III, and on 20 control bees that visited 672 flowers in total (flowers visited per bee 33.6 ± 1.4: mean ± SE) and 25 experimental bees that visited 806 flowers in total in Treatment 3 (flowers visited per bee 32.2 ± 1.7: mean ± SE) of Experiment IV.

## 4. Conclusions

The complexity of the decision process with respect to foraging that emerges in this study is intriguing. Much of the modeling for foraging theory is based on a maximum likelihood approach [79,80]. This model assumes foragers approach a new problem naively, without preconceptions about cues associated with rewards. A random sample is used to estimate the reward probability-distribution associated with different choices [49]. In a simplistic example, a bee that foraged on a patch of blue and yellow pansies at one time and later was faced with a patch of blue and yellow mums would approach the latter as a very separate problem. The foragers would take a random sample of the mums even though they have individual experience of the rewards provided earlier by pansies. 

In contrast, the Bayesian model for the same foraging problem assumes that individuals are not naive when approaching a new problem; they have some preconceived expectation of rewards associated with different cues and use preconception to make initial foraging choices [81]. The Bayesian approach is based on updating the preconceived expectations as events occur [48,49]. Here bees in the simplistic pansy/mum problem would assume that the reward distribution in mums matches that of pansies until they discovered otherwise through biased flower choices. However, many organisms, including humans, do not appear to innately use a Bayesian approach to problems [82,83]. In fact, Tversky concludes, “*man is apparently not a conservative Bayesian*: *he is not Bayesian at all*” [84].

Bees visiting blue-white flower patches clearly learned which flower color offered the great caloric reward in Treatment 2 (morning site), limiting visitation to that flower color. Further, expectations learned in the morning were used to choose flowers to visit in the afternoon site, even when floral morphology differed greatly. The maximum likelihood model clearly fails to predict observations. Further, our findings dovetail with those of Naug and Arathi [50], where honey bees visiting a single-site updated reward information in a Bayesian-like manner. Honey bees may use a Bayesian approach to problems and apparently do so at least under some conditions.

Nevertheless, our results show that when presented the same problem but with blue and yellow flowers the foragers did not follow a Bayesian approach. Neither did they use a maximum likelihood approach. Here bees receiving a much lower caloric reward fail to act on information that would maximize net caloric gain in Treatment 2 (morning site). Further, foragers maintained their individually based flower color constancy when approaching the new problem at the afternoon site, even when faced with different floral morphology and odor. This may correspond to Waite’s description of heuristic rules that are not rational in terms of economics and do not follow either Bayesian or maximum likelihood models [85]. Thus, the cognitive architecture used in foraging could be more complex than either the Bayesian or maximum likelihood models predict, although these models may be predictive under some contexts. Still, another dimension of complexity exists since aversive conditioning and walking from flower to flower are known to break the blue-yellow flower constancy described (e.g., [72,86]), and lead to potentially other approaches to novel foraging problems.

Our experiments were not designed to test for carryover of working memory or speed of decision making. Honey bee working memory holds information on flower pattern for up to 5 seconds before the accuracy begins to decrease with time delay [87], while information on the most recently visited flower color influences subsequent color choices for up to 1–2 min. In our experiments, the memory for color was still part of the search strategy after a minimum 5 h time lag time when using both flower color dimorphism.

### 4.1. Blue-Yellow Dimorphic Flower Patches

Free-flying bees visiting blue-yellow dimorphic flower patches did not visit flowers randomly with respect to color, even initially. Rather, some bees showed fidelity for yellow flowers and other bees for blue flowers, as has been previously described for one-site color choice tests [38,45,47,52]. Further, when flowers offered differing rewards in Treatment 2, individual fidelity to floral color did not change. This color-based context-dependent behavior is also observed when costs rather than benefits differ between blue and yellow flowers [45,47], and appears to even be a response of honey bees that have never foraged [55]. In this study, the flower color fidelity of a forager in the morning flower patch carried over to the afternoon patch, despite a minimum time lag of 5 h, even when flower shape and odor, in addition to the location and time, differed from flower patches in the morning. These results support the model developed over the last 30 years (e.g., [38,39]) to explain the nature of “individual constancy” foraging in honey bees. The cue learned to associate color with reward in one environment was retained by the same individuals foraging in a new environment.

Honey bee colonies shift their foraging force among food sources even within the same day to match the temporal productivity of different floral resources (e.g., [88,89]). Circadian-clock control was originally hypothesized to account for the *en masse* arrival of the crop attached forager just when floral resources become productive each day (e.g., [90]). Controlled experiments, however, show that a very limited number of bees monitor a site at any time of the day, but when the site becomes productive the monitoring bees communicate that information rapidly throughout the colony via the nectar’s odor [91,92]. Indeed, by simply injecting a puff of the scent associated with the nectar into the hive elicits *en masse* re-recruitment of foragers to a site [54,93] in what Reinhard calls “scent-triggered navigation” [94]. Indeed, monitoring has been shown to occur all day long for a site with just a 2 h afternoon rewarding period [76], which contradicts what would be expected from circadian-clock triggered foraging. 

Only recently have we realized that foragers monitor multiple sites, even when the productive period of some sites overlaps [76]. There appears to be a dynamic system of eventual loss of non-rewarding sites from memory, monitoring of several temporally rewarding and recently rewarding sites where an individual foraged previously, and addition of newly rewarding sites to the current repertoire of an individual’s active memory of foraging locations. Monitoring a site is in itself a dynamic system where different members of the site’s foraging group are monitoring it in any specific time-block or even day [76]. Here we add to that view of the honey bee by examining how a bee approaches foraging at a new site. In our study bees used CS (conditioned stimulus) information from other sites they have visited earlier in the day. They did not take a random sample of flowers as in a maximum likelihood approach to a novel problem. Rather when visiting blue-white dimorphic flower patches they used existing memory information from another location to make initial flower visitation decisions, which is a Bayesian-like approach to novel problems. 

The ability to monitor multiple sites has been explained by honey bee use of independent time-linked memory gestalts [90], which would allow an individual to forage differently on temporally over lapping foraging sites. The same type of gestalt argument has been used for prey recognition in a number of insect predators, and not fared-well under experimental scrutiny (e.g., [95]). Of course the present study suggests that in some sense any new situation is perceived initially as a continuation of what a forager was previously visiting; that is the basis of Bayesian learning. However, the artificial flower patch does not appear to be remembered as a gestalt image by a honey bee. For instance, if the patch consists of blue flowers smelling of clove as against yellow cinnamon-scented flowers, any given bee will visit many flowers of just one of those color-scent combinations. If scents associated with the colors are switched, some individuals remain constant to the original color while others are faithful to the original odor, shifting their attention to the new color [96]. This suggests that the flower patch is not a gestalt image but rather a collection of individual CS cues where individuals pick out a primary CS. 

Using a fixed, single rewarding cue [90] rather than different simultaneous rewards [52] fundamentally changes the problem for foragers. This change leads to geographic point-location recognition as the primary cue, and can be used to regulate the number of recruits to experimental flower patches just a few meters away [51]. However, even when forced to visit only blue or only yellow flower by giving them no option, honey bees revert to their initial color constancy when again given a choice on a blue-yellow flower patch [52]. Of interest now are questions of secondary cue use when the primary CS does not exist in a new site (e.g., [73]).

The ephemeral nature of working memory [87], when combined with our previous observations of innate responses of honey bees that have never foraged [55], support the suggestion of Brown [97]; honey bee tendency to maintain fidelity to color is not simply a product of learning, at least when dealing with blue-yellow dimorphisms. This phenomenon has been shown to occur under a wide range of experimental conditions with honey bees [38,39,45,47,51,52,98]; however, this color-context flower constancy has not been reported in experiments using bumble bees, and appears to mark a fundamental cognitive architectural difference from bumble bees [70,99]. Further, there are studies on the foraging ecology of the honey bee using yellow and blue flowers that have not reported this blue-yellow constancy (e.g., [37,39]), but these studies do not necessarily conflict our results. Their experimental setting used flowers that allowed the bees to walk between color types and, according to Menzel and Erber [100], Opfinger realized as early as 1931 that the bees learn color cues just before they alight on flowers. The experimental setting in our study used pedicellate flowers forcing the bees to make decisions while inflight approaching flowers; switching from pedicellate flower to a bee-board design results in a change in honey bee foraging behavior [72]. Color reverse learning experiments show that bees will change flower fidelity under some other conditions [86]. As we show here and in past experiments rewards alone do not appear to be sufficient [38,39,45,46,47,51,52] but when combined with an aversive stimuli (e.g., water in unrewarding flowers [101]) can elicit change in blue-yellow flower constancy [86]. Although afternoon flower choice was predicted from morning flower-fidelity when using blue-yellow flower patches neither a Bayesian nor a maximum likelihood problem approach was being used by foragers since no learning occurred with change in rewards in the morning.

### 4.2. Blue-White Dimorphic Flower Patches

In contrast to the behavior seen on blue-yellow flower patches, bees visiting blue-white patches visited flowers randomly with respect to color in the morning when rewards did not differ between flower colors. When presented flowers that differed in reward in Treatment 2 in the morning, bees readily learned to restrict visits to the flower color offering the greater reward, whether white or blue, thus maximizing energy resources. This behavior has been reported for bees in one-site choice tests when costs [47] as well as benefits [43,52,53] differ between these flower colors. However, here we saw that bees retain their morning search strategy when approaching a novel situation in the afternoon at a completely different foraging location, even when flower shape and reward odor suggest alternative flower species are being visited. 

Visiting two or more foraging locations in nature each day seems to be common [102], and bees can be trained to do so at specific locations (see [103]). This foraging flexibility allows honey bees in a changing floral reward landscape to change preferred floral-like patterns associated with rewards simultaneously with time of day and task [104]. Further both honey and bumble bees make short flights when encountering rich nectar resources [105,106], and foraging sites as close in proximity as 10–20 m are treated as distinct [107].

Even though floral color is well-established as the primary cue used by honey bees in discrimination tests (e.g., [98,108,109]), individuals are capable of detecting flower-like patterns in parallel with color [109]. Further, Pahl *et al.* [102] found that honey bees use memory of floral color and/or shape along with time of day to make foraging decisions in a new location. 

Our data show that bees do not limit memory use to a specific space and time. Since foragers showed learning between treatments, the blue-white dimorphic flower patch data suggest that bees use a Bayesian like approach to novel foraging situations.

### 4.3. Angiosperm Evolution

The results we observed reflect pollinator activity in some natural environments where bees confuse flower species based on floral color similarity [30,51,110,111]. Confusion in many cases involves Angiosperm evolution to deceive pollinators [112]. This pollinator deception by Angiosperms now appears to be a phenomenon more common than originally thought, and is utilized by at least 7,500 species [113]. 

Floral deception may succeed because plants are able to exploit bees that have not learned which species do not produce rewards. However, this explanation poses a dilemma as long as flower discrimination is essential for optimal exploitation of food sources [80,114]. An alternative explanation is that pollinators do not need to be naive to be deceived. Flowers are not always reliable food resources due to timing of flower opening and closing, and due to prior visitation by pollinators [113]. Thus, costs incurred due to learning flower differences in great detail are a disadvantage to this strategy [8,24,32]. In fact, foragers exposed to rich floral environments may benefit by focusing on a single flower cue, rather than on a suite of cues that more closely discriminate among flower species [115,116].

Bees in this study treated memory information as initially applicable to all situations regardless of time or location. They did not approach different locations as new problems, and their first response was to base foraging on previously learned relationships until information to the contrary was incidentally gained. This approach to foraging problems, rather than an energy maximization approach with strictly linked time and spatial memory, facilitates the evolution of angiosperm deception.

Selection might favor rapid forgetting in foragers that exploit short-term, spatially unpredictable resources, but favor lasting memory retention in foragers exploiting long-term predictable resources [9]. Despite the ephemeral duration of a flower and the uncertainty in nectar rewards linked to individual flowers, blooming plants can be resources that are temporally and spatially predictable in the same environments that support honey bees. Plants are fixed to a location in a floral landscape and each plant produces many flowers through its season. Menzel [117] suggests that flower memory is linked to spatial memory. Thus, lasting memory retention of flower cues can be linked to the more inclusive selection for spatial memory of flower patches. Some plants exploit this lasting memory since deceptive plants can rely on a pollinator’s lasting memory of a similar flower color previously learned to be rewarding. 

Memory consolidation in bees may be an adaptation to exploit the floral landscape [100,118]. Menzel [118] made reference to a threshold value of three flowers for long-term memory formation, and in our experiments each bee visited between 30 and 50 flowers in each treatment. The energetic costs of foraging guided by a color search image will depend on the rate of environmental change. If the rate of change is low, and the likelihood of finding the same flower species in the floral landscape is high, then foraging with the color image is economical. Even if “mistakes” occur (mimics, or flowers that have been emptied by other foragers), they are diluted by the prominent presence of the target plant. However if the color image becomes an anachronism due to a high rate of environmental change, costs of floral constancy to color during foraging will be apparent. Thus, observed behavior may reflect the honey bee’s common environment. 

### 4.4. Agricultural Settings

The behavior of foragers we observed also has some similarity to that reported in agricultural settings. Agricultural work with honey bees has shown foragers to become “*crop attached*” [119,120]. That is, each forager restricts its visitation to one flower type, which was originally thought to be a reflection of environmental patchiness. However, attempts to produce hybrid seed between cultivars failed even when large numbers of bees were placed in relatively small cages with two flower-color variants of alfalfa crowded together [59]. Bees visited both cultivars extensively, but few foragers visited both, and only 2% of the seed was hybrid. Similar problems have been experienced with Brussels sprouts and kale [60,61], and in natural communities of plants (e.g., [121,122,123]). 

Crop attachment may be a foraging alternative strategy to energy maximization for honey bees. Once in this mode, sampling ceases as long as an “acceptable” rate of reward is obtained. Time between morning and afternoon patch productivity may be important in this switch to a crop attached foraging mode since honey bee foragers can clearly learn to switch flower color fidelity several times in an experiment performed at one time if they are not constrained by color options, or if rewarded color choices are presented singularly [45,47,52]. This may reflect memory consolidation of cues and a lack of memory of the reward specifics.

Of course, honey bees are not the only agriculturally important pollinator or even always the most effective (e.g., [124]). Further, research over the last decade highlights substantial differences in foraging behavior even between closely related bee genera (e.g., [70]). Of agricultural interest here is the fact that bumble bees are considerably less constant, often visiting several flower species in one foraging bout [30,46,70].
